# Exploring care quality in midwifery clinical practice settings in Ghana – a qualitative study

**DOI:** 10.1186/s12909-025-06861-0

**Published:** 2025-02-20

**Authors:** Herborg Holter, Anna Williams, Tochi Chidi, Moa Karlström, Fredrica Hanson, Malin Bogren

**Affiliations:** 1 Institute of Health and Care Sciences, Sahlgrenska Academy, University of Gothenburg, Gothenburg, Sweden; 2 Data, Design + Writing, Oregon City, USA; 3 Ghana Registered Midwives Association, 1st Circular Road, H/No 11B Cantonments, Accra, Greater Accra Ghana

**Keywords:** Midwifery education, Midwife students, Quality midwifery, Clinical supervision

## Abstract

**Background:**

High-quality care is a significant factor in reducing maternal and neonatal mortality. There are known barriers affecting midwives’ ability to provide quality care in low- and middle-income countries. The presence of qualified and competent midwives, coupled with the elimination of barriers, is essential for enhancing care quality, especially in education program clinical practice settings.

**Aim:**

To explore factors that affect Ghanaian midwifery students’ provision of high-quality care while on clinical rotation.

**Method:**

Six focus-group discussions were conducted with a total of 36 midwifery students in Accra, Ghana. Data were analyzed using deductive content analysis applying a conceptual framework identifying social, economic and professional factors influencing the provision of high-quality care.

**Results:**

Social factors identified patient resistance to student midwives, class-based discrimination, traditional practices being preferred over evidence-based care, communication barriers, and poor security. Economic factors were unexpected expenses and inadequate compensation. Professional factors were lack of necessary materials, insufficient number of staff, and a theory-practice gap between classroom learning and hands-on experiences.

**Conclusion:**

Factors impacting Ghanaian midwifery students’ ability to provide high-quality care in clinical settings were summarized, highlighting social, economic, and professional challenges. Key issues include patient resistance, class-based discrimination, inadequate compensation, theory-practice gaps, and lack of materials. The findings emphasize the need for improved support, resources, and quality clinical learning environments. There needs to be investment in infrastructure and prioritization of pedagogy in clinical settings to enhance midwifery education and care quality in Ghana and more broadly in low- and middle-income countries.

**Clinical trial number:**

Not applicable.

**Graphical Abstract:**

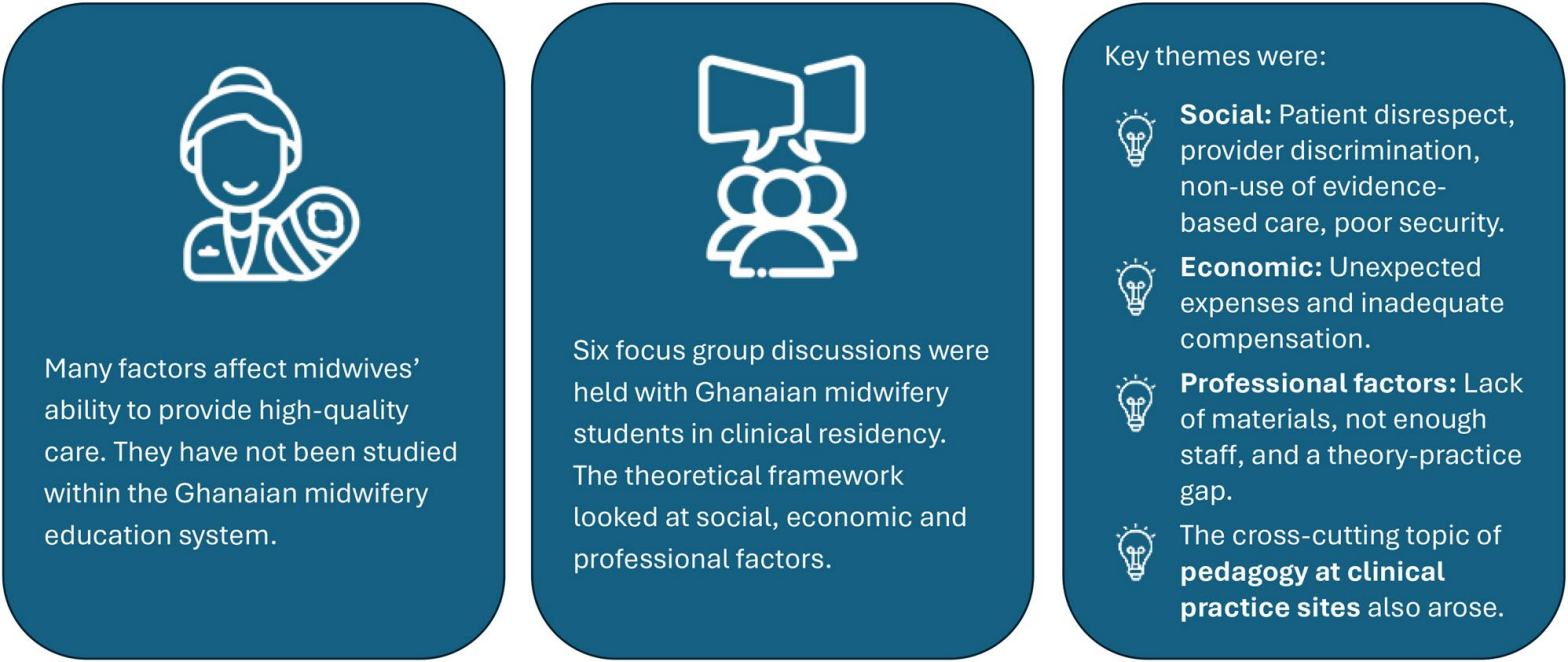

**Supplementary Information:**

The online version contains supplementary material available at 10.1186/s12909-025-06861-0.

## Introduction

Midwifery education, integrating both theoretical and practical components, is essential for developing competent midwives. Equipping students with essential competencies for midwifery practice [1] is crucial to improving maternity care globally, particularly in low-income countries (LMICs) where maternal and neonatal mortality rates remain high. However, research from many low- and middle-income countries shows a disparity between theory and practice in midwifery education [2, 3]. Significant gaps exist between global quality standards for midwifery education and their implementation [4, 5]. Evidence from sub-Saharan Africa indicates that students often struggle with inconsistent access to quality infrastructure, instruction, and clinical learning experiences, which are crucial for developing their skills [6, 7]. In particular, as the quality of care and supervision at clinical practice sites impacts students’ ability to provide competent care, educators at such sites require instruction and training to provide it [8, 9].

Systemic challenges within health systems further complicate midwifery education and practice across low-and middle-income countries [2, 6]. Poor working conditions, inadequate compensation, ineffective policy frameworks, and limited regulation undermine the quality of midwifery education and practice. Additionally, there are few opportunities for professional development, which contributes to entrenched gaps in the health workforce [10]. These issues extend to clinical practice sites, where midwifery students gain essential hands-on experience [7].

Ghana serves as an example of these challenges. With a maternal mortality ratio of 263 per 100,000 live births, Ghana has a moderate to high maternal mortality ratio [11]. Despite 87.6% of births being attended by skilled healthcare personnel, barriers to accessing care and limited use of evidence-based interventions continue to affect the quality of care. Progress across the country has been heterogenous with ongoing challenges related to infrastructure, human resources, and essential medicine access [12]. Resource constraints create challenging work environments and lead to burnout among maternal healthcare providers [13]. Midwifery students in Ghana often experience high stress levels and receive limited supervision during their clinical placements [14, 15]. Research has documented that preceptors—experienced midwives who guide new midwives in clinical practice through meaningful learning experiences—may be unavailable to perform this role, lack compensation for the work it entails, and may not have guidelines and policies detailing the role’s requirements [16, 17]. In addition, not having a governance document that defines specific quality assurance practices, such as routine meetings and staff evaluations, results in weak or non-existent quality assurance systems governing some midwifery training institutions in Ghana [18].

Understanding the barriers to quality midwifery education is essential for addressing broader challenges in maternity care. This is especially relevant in settings where poor infrastructure and resources strain the healthcare system [19, 20]. Although research exists on midwifery education in Asia [2] and sub-Saharan Africa [3, 5, 6], the literature focused on Ghana is still nascent. Most relevant papers focus on sub-national regions or fold midwifery education topics into nursing education research and fail to provide adequate detail on midwifery specific issues [13, 15, 18, 21, 22].

The aim of this study is therefore to explore the factors affecting Ghanaian midwifery students’ ability to provide high-quality care during their clinical rotation. By identifying these factors, decision-makers can be better informed when considering options for strengthening midwifery education programs. Ultimately, improving midwifery education is crucial for building a strong workforce capable of supporting better maternal and newborn health outcomes.

## Methods

### Design

This was a qualitative study that used focus group discussions (FGDs) with midwifery students in three public midwifery colleges in the Greater Accra Region. Data were analyzed using a deductive approach in alignment with Elo and Kyngäs [23]. The study received ethical clearance from the Institute of Health and Caring Science Ethical Committee at the University of Gothenburg, Sweden. The data are reported according to the COnsolidated criteria for REporting Qualitative research (COREQ) checklist [24].

### Conceptual framework

**Fig. 1:**
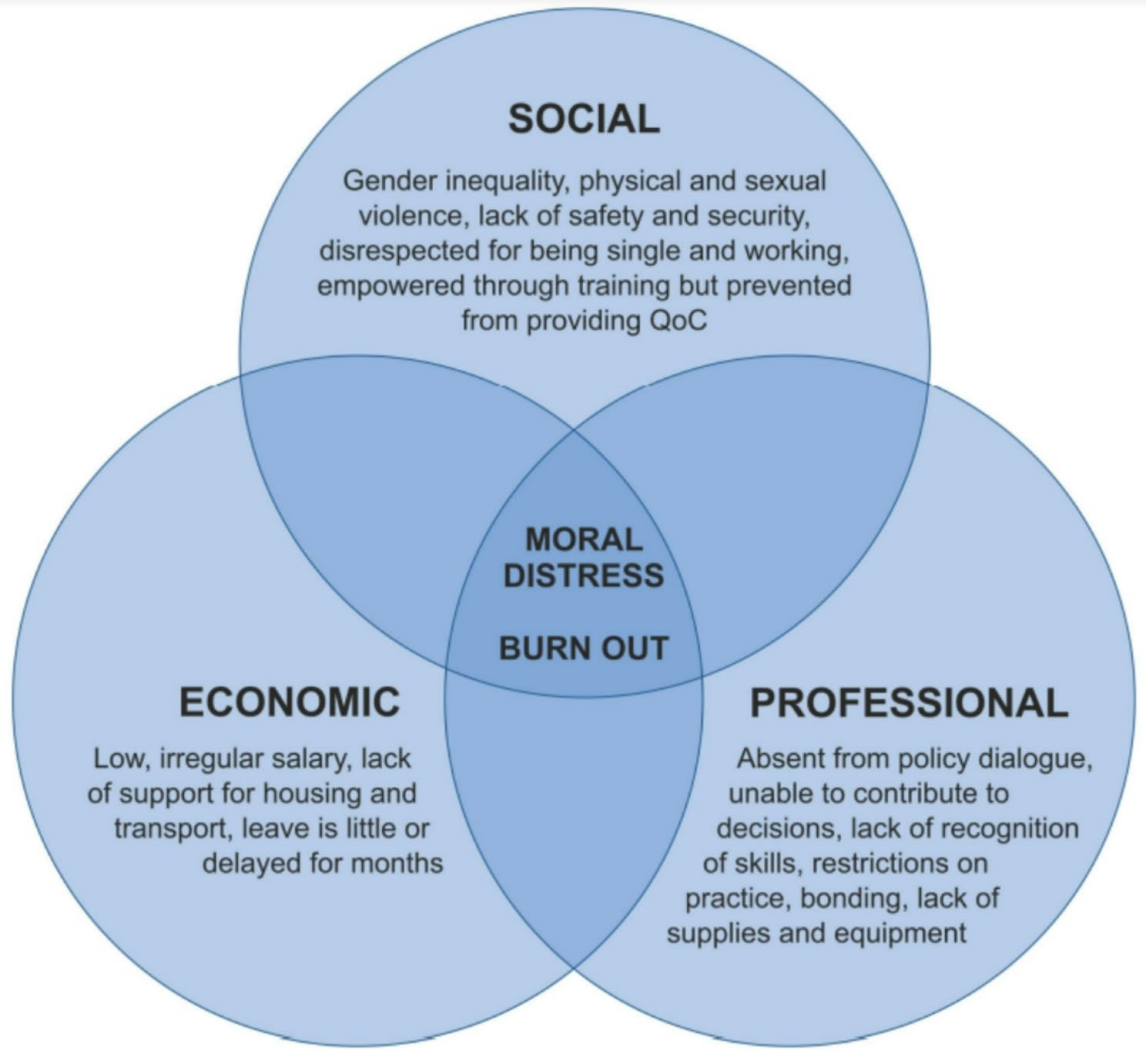
From Filby A, McConville F, Portela A [10]

The data collection and analysis was conducted according to Filby et al.’s conceptual framework describing social, economic and professional barriers to quality midwifery care [10] (Fig. 1). Social barriers refer to the culturally constructed context of childbirth, along with gender inequality and lack of female empowerment. Economic barriers comprise the absence of wages, informal payments, and insufficient government funding. Professional barriers include inadequate staffing and regulations, poor working conditions, weak facility management, lack of affordable transport, and insufficient investment in quality midwifery education. These barriers come together resulting in moral distress and burn out across the profession, amplifying care quality challenges.

### Setting

Ghana has 41 state and 18 private midwifery colleges. There are three different midwifery education programs in Ghana; (i) a direct entry four-year program; (ii) a direct entry three-year diploma program; and a (iii) two-year post basic program. The programme maintains a balanced approach with 50% theoretical studies and 50% clinical practice [25].

### Data collection

Data were collected in November 2023. A total of 519 second- and third-year midwifery students were invited to participate. Participants were recruited with the assistance of a senior midwife involved in the Ghana Registered Midwife Association. Inclusion criteria were midwifery students enrolled in any nursing and midwifery training colleges in Accra, Ghana, specifically those in their second or third year of study. Exclusion criteria included nursing students and midwifery students in their first year. The exclusion of first-year students was based on not having commenced their clinical practice placements during the interview period, meaning they lacked clinical experience in the field of midwifery. Formal letters seeking approval for midwifery students’ participation were sent to three nursing and midwifery training colleges: all three granted approvals. Teachers asked their respective classes for volunteers to partake in FGDs when the researchers arrived at the colleges.

A total of eight FGDs were conducted; however, two FGDs with six participants each were excluded due to interruptions resulting in short discussion duration. Among the remaining six FGDs, four had six participants, one had five participants, and one involved seven participants, resulting in a total of 36 participants. All participants were female and fell within the age range of 18 to 35 years.

Verbal and written consent were gathered from all participants before data collection. Participants were encouraged to speak freely and reassured they could withdraw at any time. Each FGD lasted between 26 and 47 min with an average of 34 min. Discussions took place in English in a private room. Recorded interviews were anonymized and assigned numerical identifiers accessible solely to the authors.

A semi-structured interview guide with open-ended questions adapted from Bogren et al. [26] was used to explore factors affecting the provision of high-quality care from a social, economic and a professional perspective. For details, see Supplemental Material. FGD recordings were transcribed using Microsoft Word’s transcription function. Subsequently, recordings were listened to twice and necessary changes were made to ensure transcription accuracy. To maintain anonymity and de-identification of the participants, the transcripts were not shared with the participants for comments or correction.

### Data analysis

Transcripts were read and reread to develop familiarity with the data. Meaning units were identified that aligned with Filby et al.’s conceptual framework describing social, economic and professional factors that affect quality midwifery care [10, 23]. Meaning units were compared for similar content and coded. Codes were categorized into emerging sub-categories and then the overall categories of social, economic and professional factors influencing provision of high-quality care. The initial analytical process was completed by TC and MK jointly, with repeated discussions until full agreement was reached. All authors followed this analysis process and approved the final version. Table 1 provides an example of the analysis process.

**Table 1:** Flowchart of examples from the analysis process on factors affecting Ghanaian midwifery students’ ability to provide high-quality care during their clinical rotation

Meaning units	Condensed meaning unit	Code	Sub-category	Category
But sometimes we do discriminate and then think people are rich and others are poor and then we give bad treatment to others. Meanwhile, they all deserve the same treat… you know, yeah…. And it’s very bad	Sometimes we discriminate and give poor treatment to poor people	Different care depending on affluence level	Class-based discrimination	Social factors

## Results

**Fig. 2:**
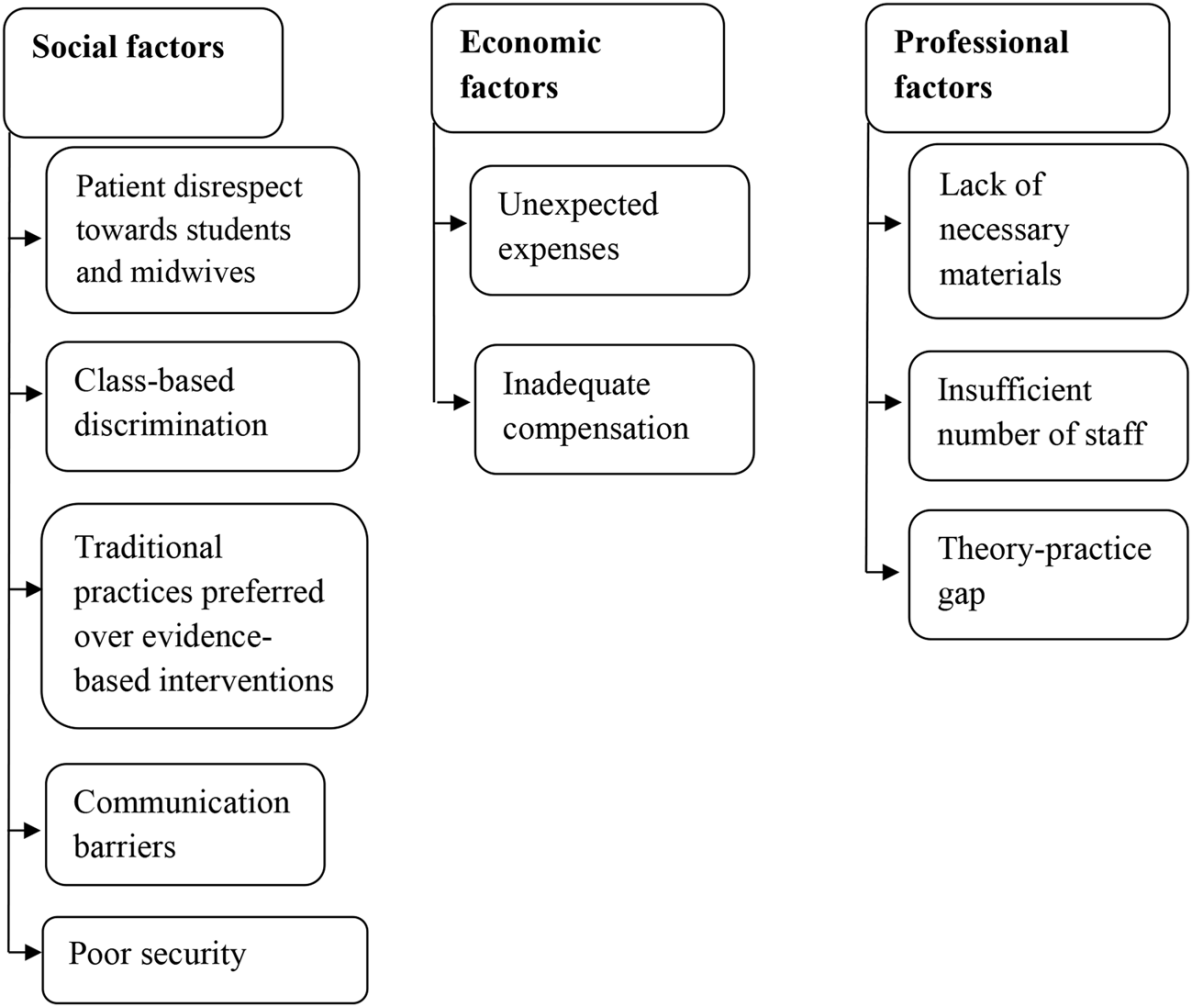
Categories and sub-categories illustrating social, economic and professional factors affecting Ghanaian midwifery students’ ability to provide high-quality care during their clinical rotation

Factors affecting Ghanaian midwifery students’ ability to provide high-quality care during their clinical rotation are presented in three categories–social, economic and professional factors–each with sub-categories; see Fig. 2.

### Social factors

These sub-categories reflect the social factors that affected the quality of care given: *Patient disrespect towards students and midwives*,* class-based discrimination*,* traditional practices preferred over evidence-based interventions*,* communication barriers and poor security.*

### Patient disrespect towards students and midwives

Some patients were resistant to being cared for by student midwives, showing a negative attitude and disrespectful treatment. In such cases, patients would request that students bring their supervisor to provide care, even though they were close to graduation.

And will be like go and call your in-charge, they [the patient] don’t want you [the student] to take care of them. - FGD3.

When this occurred, student midwives felt demotivated, and were less likely to engage positively and proactively with these patients.

Yes, and sometimes too when you [the patient] are disrespectful to them [the midwives], they [the midwives] don’t really take care of you […] Yes, so they will leave you. - FGD3.

### Class-based discrimination

Midwife students reported that patients who were well-dressed and perceived as affluent received better treatment than those whose appearance indicated economic disadvantage.

Sometimes we […]discriminate and […]think people are rich and others are poor and […]give bad treatment to others [the poor]. Meanwhile, they all deserve the same treat [treatment]. […]It’s very bad. - FGD1.

### Traditional practices preferred over evidence-based interventions

Cultural beliefs among patients around the value of traditional birthing practices emerged as playing an important role in influencing care quality. These beliefs were identified as potential obstacles to implementing evidence-based care. Patients tended to prefer traditional practices, despite midwifery students explaining their risks and the benefits of evidence-based practices.

[…]Our parents and grandparents and […] most of the things that they believe is natural to them and they used to do […] For an example […] so before labour, some of them use herbs […] and you told them not to do it and they feel like everybody does it, that’s what makes the labour easy for you. They don’t understand the reason. - FGD4.

### Communication barriers

The inability to communicate with a patient who did not speak any of the major languages in Ghana was viewed as a significant obstacle to delivering high-quality care. Communication barriers impacted the students’ ability to deliver care by hindering their interactions with their patients. Language differences, cultural misunderstandings, and limited opportunities to practice effective communication skills in clinical settings created challenges in understanding patients’ needs, providing clear instructions, and building trust. This challenge was even more pronounced when no interpreter was available or when the interpreter failed to convey the message accurately.

And when you don’t get [understand] someone. It’s quite difficult to relate with them [the patients], they feel like maybe you are [… ]not giving them the care they want, but like it’s the language barrier, that is preventing […] - FGD3.

And sometimes even if you’re lucky, to get a translator to come in […] The person might not be really translating exactly what you said to the person. And then a whole different understanding will be given to the client. - FGD5.

### Poor security

Failing safety nets caused poor security and a sense of unsafety for midwifery students. As future midwives, concerns regarding their safety during commuting to and from work were preeminent. It was considered crucial to commute in pairs, particularly during late hours due to constant fear of being threatened and robbed of their personal belongings. Indirectly, the risk of theft, violence, or harassment further undermined both the students’ learning experiences and the overall quality of care provided to patients.

Sometimes you don’t feel safe because when you get a night shift and then you are going, maybe you are […] the only one going, you see you will be afraid. […] you […] can’t go with your mobile phone because you […] are thinking you might meet armed robbers who will even attack you and then steal the phone. Not the phone alone […]- FGD1.

A student described a situation where someone, having experienced the loss of a loved one during childbirth, directed their anger towards midwives. In some cases, midwives were threatened for being midwives.

What we do is that after work, we don’t wear our uniforms home. We change in the wards. And then we move out. Yes because there are some instances whereby you are in your uniform on the roadside. Because of someone’s experience […] with a midwife. The person takes her anger on you. - FGD4.

### Economic factors

These subcategories reflect the economic factors that affect the quality of care given: *Unexpected expenses* and *inadequate compensation.*

### Unexpected expenses

Unexpected education expenses arose that influenced students’ learning process, and, in turn, care quality. Costs included a rise in school fees, cellular data for online studies, gloves, and towels. As students lacked funds for these items, the associated financial stress adversely affected their concentration during both studies and clinical practice placement.

So when this happens, where should I go and bring the money? You see? It will even affect my studies because I’ll be thinking about it and it will affect my studies. So all these things, it doesn’t help us […] the things that you bring before they will sign your evaluations for you are gloves, big tissue the large size […] - FGD1.

### Inadequate compensation

Midwifery students are assured a monthly allowance by the Ghanaian government to assist with school fees and clinical placement transportation costs. However, students shared that it was not provided, which forced them to incur more expenses than anticipated.

We don’t get any allowance, so imagine you are in the house and you are supposed to be going for clinical for a month. You use your own money for transportation […] Because there is no allowance, nothing. - FGD2.

Another economic factor that affected the care given to patients was insufficient salary, given that the cost of living surpassed their anticipated income. Compounding this issue was the recurrence of salary delays, sometimes spanned over several months. These challenges forced the midwives to seek additional sources of income such as secondary employment or initiating small businesses. Moreover, a conspicuous number of midwives opted for opportunities to work outside of Ghana where salaries are comparatively higher.

Most of […] the salaries too, they don’t come on time. And then the salary […] is nothing to write home about. Midwives’ salaries are nothing to write home about. - FGD4.

### Professional factors

These subcategories reflect the professional factors that affect the quality of care given *Lack of necessary materials*, *insufficient number of staff* and *theory-practice gap*.

### Lack of necessary materials

Lack of necessary materials adversely impacted the quality of care provided as it forced students to perform procedures without the necessary protective gear.

So how are you going to do? You see you have to use your bare hands. It’s very dangerous. What […] if the person is having maybe HIV, other infections. It’s not easy and […] we eat with the same hands too, so it doesn’t help us. […] We feel very bad. You see you will feel unsafe because you don’t know […] // Because we have families at home, so that you go home, you touch them and stuff so […] - FGD1.

The shortage of disinfectants led to their being diluted with water to extend use. Lacking towels, staff were compelled to dry their hands on their uniforms, raising infection prevention concerns. Students also reported using their hands or IV lines instead of tourniquets when taking blood samples.

It’s not easy, and when you are being asked as a student midwife, when a colleague tells you, […] perform this procedure, […] you can’t tell him. “No, I won’t do it”, you will do it because of fear. Meanwhile you are not protected, and you have to do it […] Cause you have been […] asked to […] remove […] a catheter for somebody. There are no gloves. - FGD1.

In addition, there was generally a lack of basic items and supplies, including beds, bed sheets, fans, disinfectants, towels and tourniquets. Patients were often required to provide their own bed sheets and in circumstances where beds were unavailable, care was administered on mattresses placed on the floor.

They [the facilities] have mattresses, but they don’t have […] beds […] We call them floor patients. - FGD3.

### Insufficient number of staff

The students expressed concern that the insufficient number of staff had an adverse impact on care quality due to the heightened workloads born by stretched-thin staff.

And I think that the patient, […] don’t get the adequate care they’re supposed to get because ideally I think it should be […] one patient to one […] midwife, so that the care rendered to the patient would be enough. But this is a situation whereby I’m the only person working on, […] three people. And then I won’t give you the attention I’m supposed to give you. Because I have to […] divide. […] I think when we have enough staffs, it will also help in, let me say, reduce the workload and then to reduce let me say death. Because if I don’t give you the attention I’m supposed to give you, I can cause an injury or something may happen and then it will be a problem… - FGD5.

The heavy workload led to negative attitudes towards work. On occasion, the obligation to work overtime arose when an incoming midwife intentionally arrived late to her shift.

[…] We do three shifts. We have morning, afternoon and night shifts. So because of lack of personnel some […] have to work overtime. And sometimes too, because of our bad attitude towards work, some of us don’t come on time to relieve our friends. - FGD5.

The students expressed a concern that, as future midwives, opportunities for time off were constrained by lack of staff. They had experienced that some midwives requested leave but were not able to get time off during their preferred period or were denied leave altogether.

And then sometimes you submit a letter that maybe you want to go for a leave, but maybe the time you sent it your colleague has already sent his or her letter first. So, I think your own won’t be accepted because I think since the staffing […] are not enough. So we can’t let a lot of people go on leave. So I think you have to wait till the next section for leave. - FGD5.

### Theory-practice gap

The approaches and techniques students learned in the classroom were challenging to implement in skills labs and at clinical practice sites due to lack of equipment—such as IV piggyback setups, speculum and bag valve masks—and differences in equipment learned in the classroom and that available onsite. This forced the students to deviate from their theoretical knowledge and adapt to the clinical reality.

Sometimes you must use your bare hands to do something. That is bad. - FGD1.

During clinical practice placement, when the students were tasked with bringing specific objects they had not previously encountered, it posed a challenge because it resulted in delays in patient care. Furthermore, when attempts were made to apply skills learned, the absence of materials that matched those used in their training at school was an impediment.

We are taught how to do bed making. During our practicals it’s detailed, but when you go to the wards, the equipment or the materials we have [need] to prepare the bed for a comfortable stay of the patient is limited. Sometimes, some of them [the patients] have to provide their own bedsheets. - FGD5.

This challenge was partially mitigated, however, through students’ participation in workshops in which the necessary equipment was available.

## Discussion

This study explored the contexts faced by Ghanaian midwifery students at clinical practice sites and barriers to quality care provision. We explored these barriers through Filby et al.’s framework [10], finding various social, economic and professional factors that affect midwifery students in Ghana. Across the findings, most are corroborated in the broader literature, as demonstrated in the discussion that follows. This indicates both their relevance and their validity as they paint a picture mirrored in other low- (and sometimes high-) income settings. That the prevalence of these barriers is so common within students’ clinical practice settings in Ghana and in other countries draws attention to their broad influence on care quality and presents an opportunity to address them through targeted, strategic efforts.

Patient resistance to student midwives, class-based discrimination, preference for traditional practices over evidence-based care, communication barriers, and poor security were all social factors impeding quality care. These factors conflict with the WHO principles that healthcare should be equitable, and ensure unbiased quality, regardless of class, gender or ethnicity [27]. Provider discrimination against patients based on socioeconomic status and differential treatment due to communication barriers directly contradict this principle. Moreover, equal treatment of all patients is considered a fundamental human right and a core function within the midwife’s scope of practice [28]. Education program curriculum alignment with the International Confederation of Midwives essential competencies would ensure that midwifery education programs incorporate a human rights-based approach in which all people deserve quality care [29].

The resistance to care provided by student midwives observed in this study aligns with findings from other studies. A study in Scotland reported that only 61% of pregnant women accepted a student birth attendant, citing concerns over students’ lack of experience and supervision [30]. Similarly, research in South Africa found significant resistance to care provided by male student midwives [31]. However, it is important to note that such resistance is not universally reported in the literature, particularly for female midwifery students [32].

Research in Iran identified low awareness of evidence-based midwifery care as a barrier to quality care provision, emphasizing the need for patient education [33]. Other studies have shown that women’s healthcare decisions are often influenced by family members, particularly husbands and mothers-in-law, and that good communication—from providers and within families—is associated with individuals accessing higher quality care. This evidence highlights the importance of family education and culturally sensitive communication approaches [26, 34].

Midwifery students in Ghana reported feeling insecure during their daily commutes, fearing potential attacks. This issue is not unique to this study; similar concerns have been documented in the Democratic Republic of Congo [20], Uganda, and South Africa [10]. Holtzclaw et al. (2020) note that commute-related stress can negatively impact care quality by affecting midwives’ focus and decision-making abilities [35]. These safety concerns accentuate the need for addressing the social and security-related factors that pose a challenge to midwives’ well-being and performance.

Unanticipated expenses and inadequate compensation were economic factors that were reported to affect the learning process and care quality. These have been discussed in other studies [20, 26, 27]. Filby et al. (2016) associated midwives’ low salaries with the perception of midwifery as an undervalued profession with low social status [10]. Although this reason is not explicitly identified in this study, it could be a contributing factor. In parallel with our findings, Bogren et al. (2020) identified that low and/or irregular salaries compelled midwives to seek other forms of employment [20]. Midwives seeking opportunities to work outside of Ghana negatively impacts access to qualified midwives within the Ghanaian healthcare system and weakens opportunities to improve access to quality care led by midwives. Hence, it is essential to enhance the availability of motivated and experienced midwives by improving midwives’ salaries and ensuring midwifery students receive their allowance. Policy changes to enable timely allowance disbursement could provide the financial security needed to encourage higher numbers to join the profession [20].

The transfer of theoretical knowledge to practical skills, a crucial aspect of midwifery education, was hindered by the lack of essential equipment during the Ghanian students’ clinical practice. This deficiency not only affected the learning process but also directly impacts the quality of care provided. Multiple studies, both in Ghana and other contexts, have reported on scarcity regarding necessary materials and staff [13, 20, 26, 33, 36, 37]. The issue of staff shortages emerges as a multifaceted barrier with far-reaching consequences. Research shows that insufficient staffing creates a cascade of negative effects including a strained work environment, extended working hours, a heavy workload, and feelings of insufficiency and frustration. Moreover, these challenging conditions make it difficult for both students and healthcare personnel to themselves maintain a healthy lifestyle [35]. While ultimately a health systems issue, creating and funding adequate numbers of positions at the national level is essential to creating a sustainable health workforce that can provide quality care [38].

Overall, this study found that the common lack of certain social, economic, and professional components of students’ clinical practice experiences is both a contributor to and a consequence of poor care quality. However, it perhaps more importantly draws attention to an unexpected gap in the literature: the relationship between quality clinical practice experience and students’ future competence in providing quality care. An interesting finding was that, across students’ responses, there was no mention of supervision and mentoring for midwifery students at the practice sites. Despite being probed about learning opportunities, students did not discuss any interactions with staff midwives in which they observed, were guided, or received structured feedback to help them master skills. In contrast, students’ collective responses conveyed that their roles were primarily to be either service providers or assistants. This limited their opportunities to practice alongside and learn from experienced midwives and to build upon the skills learned in the classroom. Well-trained supervisors who form positive mentor-mentee relationships with students are the foundation of effective supervision. Ultimately, a high-quality, supportive learning environment is essential to creating competent providers [39–41].

Quality pedagogy and leadership at clinical practice sites are key factors in creating future healthcare providers who provide high quality care. However, challenges with quality instruction and supervision at practice sites in low-and middle-income countries is common due to poor supervision and mentoring [42–45]. Similar limitations have been observed in studies conducted outside Africa, including issues such as underqualified preceptors, lack of standardization in clinical procedures and evaluations, and the misuse of students as junior staff rather than learners requiring guidance [40, 46].

Previous research has attempted to draw attention to the importance of strengthening quality at clinical practice sites in low-and middle-income countries. This component of midwifery education programs requires as much investment and oversight as classroom-based learning as it is the key to bridging the challenging theory-practice gap students face. The research has suggested that global midwifery education guidance documents have overlooked this key need and asserted that more focus is required to ensure quality care and supportive learning environments [8]. In alignment with Sustainable Development Goal 3 (Good health and well-being), the *Global Strategy for Women’s*,* Children’s and Adolescents’ Health* [47], and the *WHO Framework for Quality Midwifery Education* [48], this paper further supports this call. Guidance documents and investment portfolios intending to strengthen midwifery education programs in low-and middle-income countries should expand and deepen their approach to mentorship and supervision in practice settings. Mentor and supervisor behavioral parameters, student learning objectives, and management responsibilities should be laid out for clinical practice sites, and quality assurance systems be put into place to monitor them [8]. Future research could track the impact of improved clinical education on maternal and neonatal health outcomes.

### Strengths and limitations

One of the strengths of this study was the use of consistent data collection methods, as the same questionnaire was employed across all FGDs. Additionally, careful listening to recordings ensured the trustworthiness of the findings. The volunteers represent 48 students out of 519 asked across three different schools. Without additional demographic or contextual data (e.g. academic performance, or socio-economic background), it is difficult to definitively assess whether they are representative of the larger student body. However, given the relatively small proportion of students who volunteered (approximately 9.25%), there is a potential for selection bias. For example, students who volunteer might differ systematically from those who do not in ways that could influence the findings. Therefore, caution should be exercised when generalizing results from the volunteer sample to the entire student body.

The involvement of midwifery researchers from Sweden with experience of researching in West Africa enriched the study design and analysis by offering valuable insights into the regional context. However, cultural diversity within West Africa is vast, and the researchers’ experiences may not fully capture the specific nuances of Ghana. To mitigate any unintended biases that might have arisen from the researchers not being fully familiar with the context, we invited one Ghanian researcher to ensure trustworthiness in the interpretation of the results.

## Conclusion

This study identified factors that affected Ghanaian midwifery students’ provision of high-quality care in clinical practice settings. Key challenges include social factors like patient resistance and class-based discrimination, economic issues such as inadequate compensation, and professional constraints including the theory-practice gap and necessary materials. These findings align with broader literature and underscore the critical need for enhanced support and resources in midwifery education. Addressing these factors, including the cross-cutting element of pedagogy in clinical practice settings, is essential for improving the quality of both education and care. It is the responsibility of governments and their partners to sufficiently invest in the infrastructure required for quality midwifery education programs in low-and middle-income countries and, while doing so, emphasize quality learning environments in clinical practice settings. They are essential to building a quality midwifery profession, and there is still a long way to go.

## Electronic supplementary material

Below is the link to the electronic supplementary material.

Supplementary Material 1

## Data Availability

The data sets used and/or analysed during the current study are available from the corresponding author upon reasonable request.
