# Supplementary material for: Exploring care quality in midwifery clinical practice settings in Ghana – a qualitative study
Source: BMC Med Educ. 2025 Feb 20;25:281. doi: 10.1186/s12909-025-06861-0 (PMC11843960; doi:10.1186/s12909-025-06861-0)
Supplement: Supplementary file 1 — Supplementary Material 1 [file 12909_2025_6861_MOESM1_ESM.docx]

## Interview guide

1. What factors affect you to give quality midwifery care from a social perspective? Positive and negative factors.
   - Gender equality
   - Respect
   - Safety and security
   - Empowerment

- Please describe
- Does anyone have their own experiences or experiences you have heard of, that you want to share?
- Can you please describe how?
- Can you explain why you think this happens in the Ghana setting?
- Do you agree/disagree?
- Apart from what we already have discussed, can you describe or discuss any other barriers from a social perspective, which prevents quality midwifery care, as you see it?
- Please describe, and discuss why you feel in this way?
- Please, discuss what actions could be done which could change these feelings and by whom.
- Can anyone else add something?

1. What factors affect you to give a quality midwifery care from an economic perspective? Positive and negative factors.
   - Salary
   - Housing
   - Transportation
   - Leave

- Please describe
- Does anyone have their own experiences or experiences you have heard of, that you want to share.
- Can you please describe how?
- Can you explain why you think this happens in the Ghana setting?
- Do you agree/disagree?
- Apart from what we already have discussed, can you describe or discuss any other barriers from an economic perspective, which prevents quality midwifery care, as you see it?
- Please describe, and discuss why you feel in this way?
- Please, discuss what actions could be done which could change these feelings and by whom.
- Can anyone else add something?

1. What factors affect you to give a quality midwifery care from a professional perspective? Positive and negative factors.
   - Policy dialogue
   - Decision making
   - Skills
   - Practice
   - Supplies
   - Equipment
   - Training
   - Staffing
   - Competence
   - Confidence

- Please describe
- Does anyone have their own experiences or experiences you have heard of, that you want to share?
- Can you please describe how?
- Can you explain why you think this happens in the Ghana setting?
- Do you agree/disagree?
- Apart from what we already have discussed, can you describe or discuss any other barriers from a professional perspective, which prevents quality midwifery care, as you see it?
- Please describe, and discuss why you feel in this way?
- Please, discuss what actions could be done which could change these feelings and by whom.
- Can anyone else add something?
- Before we close up this focus group discussion, is there anything more you would like to share?
- Thank you very much for your participation!
